# Dauricine Impedes the Tumorigenesis of Lung Adenocarcinoma by Regulating Nrf2 and Reactive Oxygen Species

**DOI:** 10.3390/cells14100698

**Published:** 2025-05-12

**Authors:** Waleed Yousuf, Nimra Zafar Siddiqui, Perbhat Ali, Shaoxuan Cheng, Immad Ansari, Jialiang Song, Minghe Dai, Zhiyuan Qiu, Yue Zhu, Yaowen Zhang, Shuyan Liu, Yingqiu Zhang, Zhenhua Liu, Han Liu

**Affiliations:** 1 Institute of Cancer Stem Cell, Dalian Medical University, Dalian 116044, China; waleedyousuf2015@gmail.com (W.Y.); nimra.siddiqui12@gmail.com (N.Z.S.); perbhatali@yahoo.com (P.A.); C18834183096@126.com (S.C.); sjl200101@163.com (J.S.); dmh103@163.com (M.D.); qiuyanbo1996@163.com (Z.Q.); 13358942666@163.com (Y.Z.); zyw_1105@163.com (Y.Z.); liusy@dmu.edu.cn (S.L.); zhangyingqiu.no.1@163.com (Y.Z.); 2 School of Basic Medical Sciences, Dalian Medical University, Dalian 116044, China; immadansari@outlook.com

**Keywords:** dauricine, lung adenocarcinoma (LUAD), cell growth, cell migration, apoptosis, ROS, Nrf2

## Abstract

Dauricine has been shown to possess intriguing anti-cancerous activities against various malignancies. The current study examined the inhibitory effects of dauricine against lung adenocarcinoma with cell lines and animal models. MTT assay was performed in three different lung adenocarcinoma cell lines using a concentration range of dauricine. Colony formation, wound healing, Edu incorporation, and cell cycle analysis were conducted to investigate the impact of dauricine on lung adenocarcinoma cells in vitro. Moreover, flow cytometry was performed to observe the effect of dauricine on cellular ROS levels. The expression of redox regulator Nrf2 and apoptosis-related markers was assessed by Western blot. Importantly, the anti-tumor efficacy of dauricine was studied in vivo with two lung adenocarcinoma animal models, including a subcutaneous cell line-derived syngeneic model and an inducible orthotopic *KRAS^G12D^*-driven lung adenocarcinoma model. The proliferation and migration of lung adenocarcinoma cells were significantly reduced by dauricine treatment. Flow cytometry analysis revealed that dauricine treatment resulted in cell cycle arrest at G0/G1 phases in A549, H1299, and A427 cells. Intracellular ROS levels were markedly augmented by dauricine treatment. Notably, dauricine led to the downregulation of the master redox regulator Nrf2. Meanwhile, dauricine treatment resulted in decreased Bcl-2 levels but elevated expression of BAX and cleaved Caspase 3. Finally, dauricine demonstrated significant efficacy in restricting tumor progression in both subcutaneous syngeneic and orthotopic lung adenocarcinoma models. Our results corroborate the anti-cancer effects of dauricine against lung adenocarcinoma with in vivo and in vitro analyses. Our findings also provide mechanistic evidence that links the impact of dauricine to cell cycle blockage and ROS-mediated apoptosis.

## 1. Introduction

Lung cancer is the most common frequently diagnosed tumor and is associated with massive levels of mortality due to this. Lung cancer is often identified at mature stages as it remains asymptomatic during the early developmental process [1,2,3]. Worldwide, lung cancer is one of the most common cancers diagnosed, about 11.4% of all cancers, and causes about 18.0% of cancer-related deaths [4,5,6]. Up till now, lung cancer has predominantly been found in elderly persons, with about 70% of cases diagnosed with lung cancer in people aged > 65 years and less than 3% of cases in people aged < 45 years [7,8].

Non-small cell lung carcinoma (NSCLC) is one of the major sub-classes of lung cancer, and accounts for about 85% of the total lung tumor cases. NSCLC is a heterogeneous disease that exhibits distinct histopathological features and genomic subtypes [9]. It is split into four distinct categories: adeno-squamous carcinoma, large-cell lung carcinoma, adenocarcinoma, and squamous carcinoma [3]. Among these, lung adenocarcinoma (LUAD) is the most prevalent category [10,11,12,13]. The treatment approaches for LUAD include surgical intervention, chemo/radiotherapy, immunotherapy, and targeted drugs. However, it has been claimed that a high proportion of diagnosed patients do not qualify for surgery [14]. The prognosis of LUAD at early stages is affected by certain factors, including resistance to radio/chemotherapy and aggressive relapse [15,16,17,18]. Of note, drug resistance remains a significant challenge, which needs to be tackled to improve the treatment of distinct classes of lung cancer [19]. Therefore, exploring alternative therapeutic options that are safe, affordable, and effective is an urgent need in expanding our arsenal against LUAD [20,21].

Kirsten rat sarcoma viral oncogene homolog (*KRAS*) mutations are one of the most frequently observed genetic alterations in NSCLC, occurring in approximately 25–30% of LUAD cases [22]. These mutations are associated with smoking and non-smoking related lung cancer. In smokers, *KRAS* mutations predominantly exhibit cytosine-to-adenine nucleotide transversions, while in non-smokers, *KRAS* mutations often involve guanine-to-thymine transversions [23,24]. These differences in mutation suggest different oncogenic pathways, which contribute to cell survival, proliferation, and drug resistance, and may influence responses to therapies [25].

Despite being discovered early as oncogenic drivers in lung cancer, most KRAS mutations are still not treatable due to the lack of inhibitors. Lately, the development of KRAS G12C inhibitors, sotorasib and adagrasib, has shown promising results in clinical trials, which offer new therapeutic options for NSCLC. However, acquired resistance remains a major problem [26]. Tumors can bypass KRAS inhibition through alternative pathway activation, epithelial-to-mesenchymal transition (EMT), secondary mutations, or the upregulation of compensatory survival pathways, thus leading to failure of treatment [27,28]. Therefore, the urge of novel therapeutic strategies as an alternative regimen is needed to target KRAS and other resistant oncogenic drivers.

To bolster rapid proliferation, cancer cells continuously alter their metabolic requirements, allowing them to survive and rapidly flourish under various stressors [29,30]. Tumor proliferation needs an important prerequisite that regulates redox homeostasis [31]. Cancer cells modify their biochemical activities, resulting in an elevated synthesis of biomolecules and reactive oxygen species (ROS) in contrast to normal cells [32,33,34]. Elevated ROS levels can support the survival of tumor cells by activating the expression of transcription factors like nuclear factor erythroid 2-related factor (Nrf2), which initiates the upregulation of cellular antioxidant defenses to avert ROS-mediated damage, ultimately contributing to chemotherapy resistance [35,36].

Genetic alterations in Nrf2 and its regulators are recurring events in the development and progression of NSCLC, leading to constitutive expression of transcription factor Nrf2, which neutralizes cellular ROS, which is required to maintain the redox homeostasis and protect cells against toxic xenobiotics. These alterations have been reported to constitute approximately 25% of total genomic events [37,38,39]. Elevated levels of ROS in the cytoplasm lead to the translocation of Nrf2 into the nucleus, where it functions as an antioxidant transcription factor [40], promoting the survival of malignant cells against radiotherapy and chemotherapy [36]. Hence, targeting the tumor-protecting roles of Nrf2 is an attractive research topic in the development of therapies against lung cancer.

Dauricine is a diphenylisoquinoline alkaloid extracted from the Chinese herbal medicine rhizoma *Menispermum dauricum*, commonly known as Dauricum root [41,42]. This natural compound displays a wide range of biological effects, including the suppression of tumor cell growth and the inhibition of transmembrane ion channels [43]. Previous reported studies have suggested that dauricine exerts anti-arrhythmic, anti-bacterial, and anti-inflammatory activities. Additionally, recent investigation has shown that dauricine also possesses anti-tumor activity, with reported effects in pancreatic cancer [44], lung cancer, renal cell carcinoma [45], and colon cancer [46]. Based on these findings, the current study aimed to explore the therapeutic effects of dauricine as an anti-tumor agent against LUAD, through a series of in vitro assays on distinct cell lines as well as in vivo LUAD mouse models, including the cell line-derived syngeneic tumor model and transgenic *KRAS^G12D^*-driven orthotopic LUAD model. Moreover, the underlying mechanisms of the inhibitory effect of dauricine on LUAD were also investigated. Our results confirm the effectiveness of dauricine as a promising therapeutic agent against LUAD.

## 2. Materials and Methods

### 2.1. Cell Culture

Four non-small-cell lung cancer cells were procured: A549, H1299, and Lewis Lung Carcinoma (LLC) from the American Type Culture Collection (ATCC), while A427 cells were obtained from Procell (Wuhan, China). The RPMI-1640 medium (Gibco, Suzhou, China) was used for A549 and H1299, Modified Eagles Medium (MEM, VivaCell Biosciences, Shanghai, China) was used for A427, and LLC was grown in Dulbecco’s Modified Eagles Medium (DMEM, Gibco, Suzhou, China) in a humidified incubator (Thermo 3111, Shanghai, China) at 37 °C containing 5% of CO_2_. For growth supplements, every medium was augmented with 10% of fetal bovine serum (Excell) and 1% of penicillin/streptomycin (Seven Biotech, Beijing, China). All cells used in the study were routinely tested for mycoplasma contamination.

### 2.2. Antibodies and Reagents

Dauricine was purchased from Chengdu Munster (Chengdu, China). Anti-Nrf2 rabbit antibody (cat. no. 16396-1-AP), rabbit antibody against BCL2 (cat. no. 26593-I-AP), rabbit anti-BAX antibody (cat. no. 50599-2-Ig), mouse anti-GAPDH antibody (cat. no. 6004-1-Ig), and mouse anti-Ki67 antibody (cat. no. 28074-1-AP) were purchased from Proteintech (Wuhan, China). Tween-20 (cat. no. P1379) and DMSO (cat. no. D4540) were purchased from Sigma-Aldrich (St. Louis, MO, USA). Secondary antibodies, including goat anti-rabbit and anti-mouse antibodies for Western blotting, were infrared-labeled and obtained from LI-COR. All reagents were dissolved as per the guidelines provided by the manufacturers.

### 2.3. MTT

NSCLC cell proliferation was assessed via the MTT assays. A549, H1299, A427, and LLC cells were counted by Neubauer chamber and seeded (3000 cells/well) in a 96-well plate, followed by incubation for 24 h with distinct dauricine concentrations, i.e., 1, 5, 10, 15, and 20 μM. After treatment, 3-(4,5-dimethylthiazol-2-yl)-2,5-diphenyltetrazolium bromide (MTT) solution was added into each well of the plate and further incubated for 4 h at 37 °C in a humidified incubator. DMSO was finally added into each well to dissolve formazan from the reaction. The cell plates were shaken to mix thoroughly, and the absorbance was recorded at 570 and 630 nm using a spectrometer (Enspire 2300, Perkin Elmer, Springfield, IL, USA).

### 2.4. Colony Formation Assay

A single-cell suspension was prepared for the A549, H1299, and A427 cells for seeding (1000 cells/well) into a 6-well plate. The cells were incubated at 37 °C for 7 days and the medium was refreshed after every 72 h. Once colonies emerged, treatment was initiated with the designated concentrations of dauricine for 48 h as described above. Colony fixation was performed by incubation in methanol at room temperature for 10 min, followed by 0.1% of crystal violet staining for colony visualization as described [47]. The images were captured using a Bio-Rad imaging system and quantification was performed by ImageJ software (version 1.52).

### 2.5. Wound Healing Assay

Wound migration assay was performed as described previously [48]. Cultured H1299 and A427 cells in a 6-well plate were incubated at 37 °C until they reached full confluency. Afterwards, a scratch was generated in the middle of the well using a 200 µL sterilized pipette tip, and cells were washed before being incubated in medium containing mitomycin, and concentrations of dauricine were indicated. Wound recovery images were captured at 0, 24, and 72 h by microscopy (Leica, Suzhou, China) and quantification was conducted by ImageJ software (version 1.52 a).

### 2.6. Edu Assay

NSCLC cell proliferation was assessed by Edu assay. Cultured cells were seeded into 24-well plates and incubated at 37 °C. Indicated concentrations of dauricine were added at about 60–70% of cell confluency. Post-incubation steps were followed as per the manufacturer’s guidelines (Beyotime, Shanghai, China) and as described previously [49]. Finally, images were captured by an inverted fluorescence microscope (Olympus, Tokyo, Japan).

### 2.7. Flow Cytometry

In order to validate the effect of dauricine on cell cycle arrest, A549, H1299, and A427 cells were seeded into 6-well plates to grow until about 60% confluency. Afterwards, indicated concentrations of dauricine were added for treatment. Treated cells were trypsinized and harvested for overnight fixation in 70% ice-cold ethanol as described previously [50]. The following day, cells were washed and treated with the staining solution (0.2% Triton X-100, 100 µg/mL propidium iodide, 50 µg/mL RNase) for 30 min. Finally, samples were analyzed by ACCURI C6 flow cytometer (BD Biosciences, San Jose, CA, USA). Moreover, intracellular ROS levels of distinct lung carcinoma cells were also assessed by flow cytometry using the ROS assay kit (Beyotime, China). Briefly, cells were cultured in 6-well plates and treated with indicated concentrations of dauricine for 24 h. Post incubation, cells were washed with PBS before the addition of the ROS probe DCFH-DA at 10 µM for 30 min at 37 °C. Afterwards, trypsinized cells were washed with PBS, and resuspended in ice cold PBS in dark. Finally, samples were analyzed by the ACCURI C6 flow cytometer (BD Bioscience, USA). Raw flow cytometry data were processed with the FlowJo software (version 10.9.0).

### 2.8. Mouse Syngeneic Model

Animal experiments were carried out following the “1996 National Institutes of Health Guide for the Care and Use of Laboratory Animals” guidelines, and all the procedures were approved by the Institutional Animal Care and Use Committee of Dalian Medical University, Dalian, China. Mice were maintained in the Specific Pathogen Free (SPF) Facility during the experiments. A total of 10 female mice (C57BL/6) (age, 4–6 weeks; weight, 18 ± 2 g) were obtained from Vital River Co., Ltd. (Beijing, China). The mice were quarantined for one week before carrying out the experiment; housed at 55% humidity and 23 °C with a light/dark (12/12) cycle at the facility; and provided with access to food and water ad libitum. Ten mice were randomly divided into two groups, control (n = 5) and treatment (n = 5). Each mouse from both groups received 5 × 10^5^ Lewis Lung Carcinoma (LLC) cells suspended in 100 µL of PBS subcutaneously, into the right flanks of the mice. The next day, dauricine was injected intraperitoneally at a dose of 20 mg kg^−1^ body weight to the treatment group, while mice in the control groups received an equal volume of PBS intraperitoneally daily for 21 days. Mice body weights and tumor sizes were monitored daily during the experiment. Only one tumor was observed per mouse; none of the mice showed multiple subcutaneous tumors. Other general signs such as illness and endpoints were monitored and reviewed thoroughly throughout the experiment. None of the mice exhibited the signs of these endpoints until the end of experiment. The tumor was measured and the following formula was used to calculate tumor sizes: Tumor volume (mm^3^) = (length) × (width)^2^ × 0.5.

### 2.9. Transgenic Mouse Lung Cancer Model

Animal experiments were carried out following the “1996 National Institutes of Health Guide for the Care and Use of Laboratory Animals” guidelines, with all procedures approved by the Institutional Animal Care and Use Committee of Dalian Medical University. To establish the orthotopic lung adenocarcinoma model, transgenic mice harboring an inducible *KRAS^G12D^* mutation were treated with AAV2/6-CMV/Cre-ZsGreen virus through intratracheal injection. Two weeks later, mice in the treatment group received intraperitoneal injections of dauricine at a dose of 20 mg kg^−1^ body weight and control group mice received intraperitoneal injections of PBS. The treatments were administered at around 11 a.m. daily for a duration of 45 days. Following the completion of the treatment, the animals were weighed and lungs were collected for histological analysis to examine tumor nodules.

### 2.10. Immunohistochemistry

Tumor tissues were harvested to examine the proliferation marker Ki67 in both control and treated tumor tissues. The tissues were fixed in formalin at room temperature, dehydrated by graded ethanol gradients, and embedded in paraffin. IHC was conducted following the procedures reported previously with minor modifications [51]. Furthermore, 4 μm sections were cut from each paraffin block, deparaffinized by immersing the slides twice in xylene, rehydrated with decreasing ethanol gradients (100–50%), washed with running tap water, and incubated with 3% of H_2_O_2_ for 10 min at room temperature. The tissue was then proceeded for antigen retrieval. The slides were heated in a microwave for 5 min in antigen retrieval buffer, washed with PBS (3 times of 5 min), blocked with BSA for 30 min, and incubated with the primary antibody, rabbit-polyclonal anti-mouse Ki67 (catalog no. 28074-1-AP), overnight at 4 °C. The next day, slides were rinsed with PBS for 3 min and incubated with secondary antibody that was horseradish peroxidase-conjugated (ZSGB-BIO, Beijing, China) at room temperature for 1 h, washed with PBS (3 times of 5 min), and stained with 3, 3-iaminobenzidine (DAB) substrate chromogen (cat. no. ZLI-9018) for 1–5 min. The slides were washed and then counterstained with hematoxylin for 5 min and observed under a light microscope. Images were captured, and comparisons were made after observing the positive-stained cells in the tumor sections.

### 2.11. Western Blotting

For Western blotting, cells were lysed in the RIPA lysis buffer (10 mM Tris- HCl pH 7.5, 100 mM NaCl, 1% Nonidet P-40, 50 mM NaF, 1% sodium deoxycholate, and 0.1% SDS) as described previously [52]. Freshly prepared lysis buffer was supplemented with phosphatase inhibitor cocktail (Roche) and protease inhibitor cocktail (Sigma) following a previously established method [16]. After centrifugation at 15,000× *g*, 4 °C for 15 min to clear the lysates, protein concentrations were measured using a BCA assay kit (Pierce). Equal amounts of lysate proteins were separated by 8% SDS-polyacrylamide gel electrophoresis (SDS-PAGE) and transferred onto a nitrocellulose membrane. The membranes were then blocked with 4% skimmed milk in PBS for 1 h at room temperature and incubated overnight at 4 °C with diluted primary antibodies. The membranes were washed with PBS and incubated with infrared-labeled secondary antibodies at room temperature for 1 h. Finally, images were captured using a LI-COR Odyssey imager, and protein band intensities were quantified using the Image Studio program (version 4.0).

### 2.12. Statistical Analysis

All quantified data were demonstrated by mean ± standard error of the mean (SEM) with results from 3 independent biological repeats. Statistical difference between groups was tested by conducting Student’s *t*-test using GraphPad Prism software (version 5.01), and *p* values less than 0.05 represent significant differences.

## 3. Results

### 3.1. Dauricine Inhibits the Proliferation and Migration of LUAD Cells

**Figure 1:**
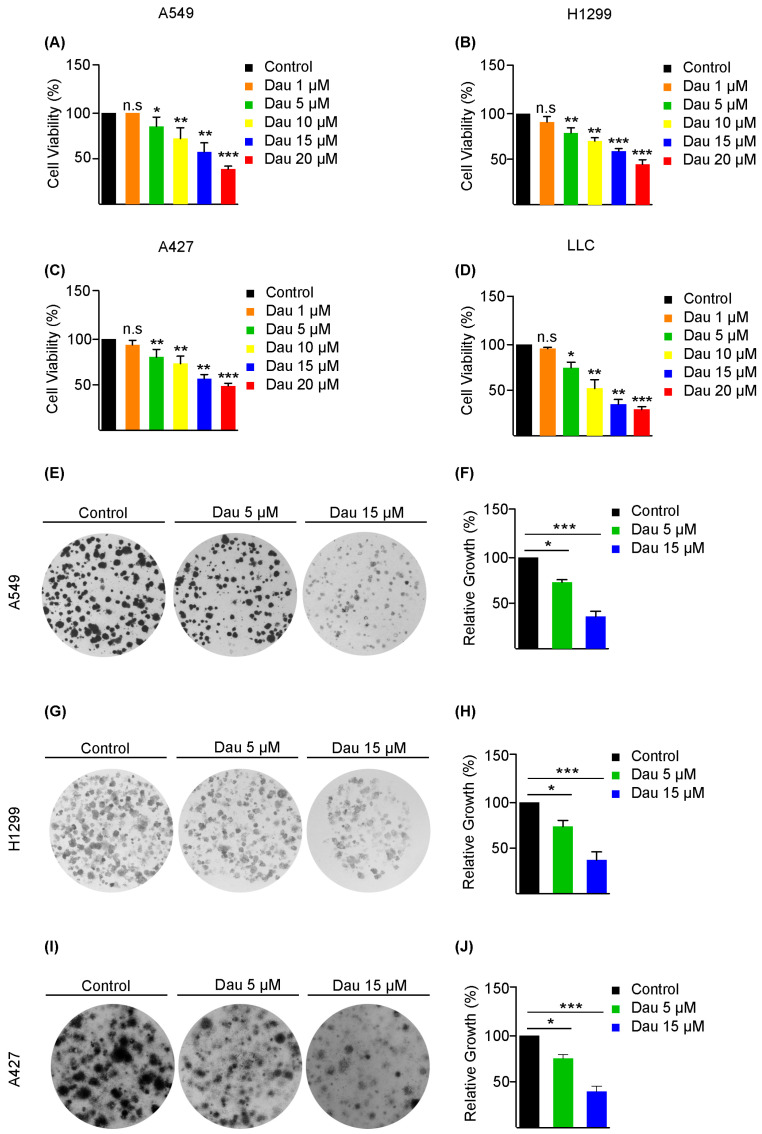
Dauricine inhibits the proliferation of NSCLC cells. Lung cancer cells (**A**) A549, (**B**) H1299, (**C**) A427, and (**D**) LLC were treated with various concentrations of dauricine (Dau) at 0, 5, 10, 15, and 20 µM for 24 h before exposed to MTT solution to assess growth progression. A colony formation assay was conducted to estimate the proliferation of lung cancer (**E**,**F**) A549, (**G**,**H**) H1299 and (**I**,**J**) A427 cells under the influence of dauricine at concentrations of 5 and 15 µM for 48 h. Post-treatment images were captured to perform a comparative analysis to determine the average sizes of colonies for each condition. Quantification was carried out using ImageJ, and the results are represented in columns; data are shown as mean ± SEM, with *, ** and *** indicating *p* < 0.05, *p* < 0.01 and *p* < 0.001, respectively. n.s., not significant.

We evaluated the anti-proliferative potential of dauricine on LUAD cells. The cell viability was measured through MTT assays. Significant decreases in cell viability were observed in A549, H1299, A427, and Lewis Lung Carcinoma (LLC) cells from 5 µM of dauricine in a dose-dependent manner (Figure 1A–D). Additionally, we examined the growth inhibition influence of dauricine on LUAD cells over a long incubation duration through colony formation assays with dauricine treatment at two different concentrations: 5 and 15 µM. We found that colony sizes were significantly reduced in A549, H1299, and A427 cells treated with dauricine at both concentrations, with the 15 µM group demonstrating greater suppression compared to the 5 µM group (Figure 1E–J).

**Figure 2:**
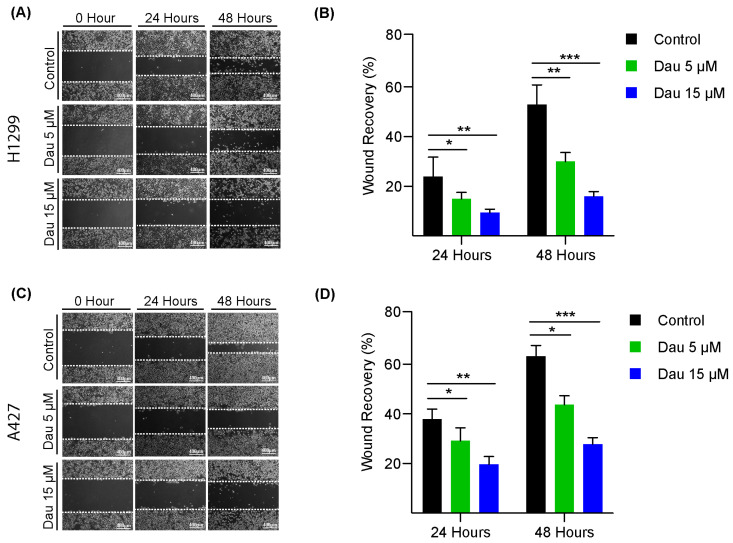
Dauricine suppresses NSCLC cell migration. LUAD cells allowed to form a confluent monolayer were processed for uniform scratching with a 200 µL tip. (**A**,**B**) display H1299 cell wound recovery micrographs (Scale bar = 400 µm) of indicated concentrations and a column chart representing the quantification of wound migration. (**C**,**D**) A427 cell images depicting wound recovery progress along with quantification shown in column graphs for concentrations of 5 and 15 µM. Image J software was used for quantification, showing means ± SEM, with *, ** and *** indicating *p* < 0.05, *p* < 0.01 and *p* < 0.001 respectively.

Furthermore, scratch assays were conducted to evaluate the impact of dauricine treatment on cell migration using H1299 and A427 cells. In the H1299 control group, which was exposed to DMSO, wound recovery was close to 60% after 48 h, while H1299 cells treated with 5 µM of dauricine showed approximately 32% migration into the wounds, and those treated with 15 µM of dauricine displayed significantly inhibited wound recovery of merely around 18% (Figure 2A,B). Meanwhile, A427 cells in the control and treatment groups (5 and 15 µM) exhibited wound recoveries of 63.15%, 43.71%, and 27.85% on average, respectively, after 48 h of incubation (Figure 2C,D). Therefore, our observations confirmed that dauricine demonstrated tumor-inhibitory effects on LUAD cells by suppressing both cell proliferation and migration.

### 3.2. Dauricine Deters Cell Cycle Regulation and Growth of LUAD Cells

**Figure 3:**
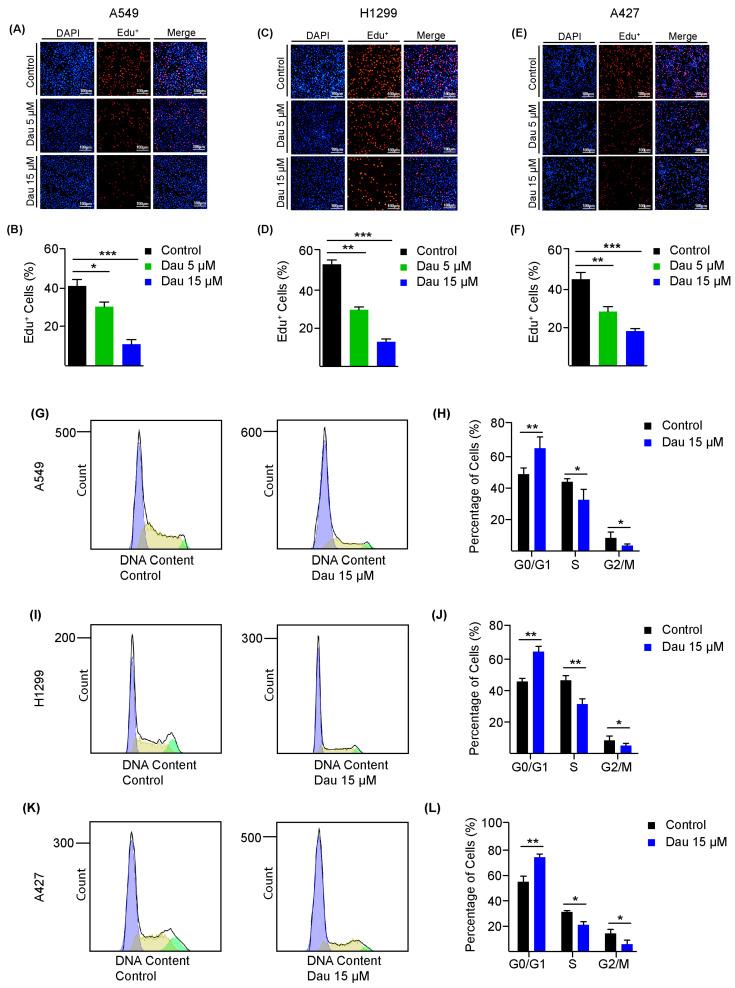
Dauricine restrains the progression of the cell cycle and regulates cell growth. (**A**) A549, (**C**) H1299, and (**E**) A427 were exposed to doses of dauricine (5 and 15 µM) for 24 h, before EdU proliferation assay was performed as shown in the images. Post-staining images were captured using an inverted fluorescence microscope, focusing on three randomly selected fields for each condition. The quantification was analyzed for (**B**) A549, (**D**) H1299, and (**F**) A427 cells through ImageJ software and is presented in column charts. Histograms of (**G**) A549, (**I**) H1299, and (**K**) A427 illustrate the distribution of LUAD cells into distinct phases of the cell cycle after treatment with dauricine (15 µM) for 24 h. The quantification of cells in different phases is displayed on the right for (**H**) A549, (**J**) H1299, and (**L**) A427 cells. All error bars represent the standard error of the mean (n = 3), with *, ** and *** indicating *p* < 0.05, *p* < 0.01 and *p* < 0.001 respectively.

We further evaluated the inhibitory effects of dauricine on LUAD cell progression, which can be assessed by synthesis and duplications of DNA during cell cycle using the Edu incorporation assays, which adopt a 5-ethynyl-2′-deoxyuridine analog along a click chemistry method. To detect actively growing cells between three comparative groups, LUAD cells were treated with DMSO as control, or dauricine at 5 and 15 µM. As shown in Figure 3A,B, the propagation of A549 cells was reduced by 10.9% and 30.62% with 5 and 15 µM of dauricine treatment, respectively, following 24 h incubation, in comparison to the control. In addition, H1299 cell expansion was hindered by 23.82% at 5 µM and 40.68% at 15 µM of dauricine treatment, as measured via Edu assay (Figure 3C,D). Furthermore, it was found that A427 cell growth was suppressed by 16.93% and 27.27% at concentrations of 5 and 15 µM of dauricine, respectively, as compared to the control group (Figure 3E,F). It has been established that cancer propagation is reflected through successive cell cycle progression. To determine the effects of dauricine on the cell cycle distribution of LUAD cells, we performed flow cytometry of LUAD cells with or without dauricine treatment at 15 µM. We found that A549 cells treated by dauricine were significantly arrested in G1-Phase after 24 h incubation (Figure 3G,H). Consistently, H1299 cell population was substantially enhanced by 18.33% in the G1-Phase, reduced by 14.88% in S-Phase, and reduced by 2.89% in the G2-Phase following dauricine treatment (Figure 3I,J). Furthermore, as shown in Figure 3K,L, the proportions of A427 cells were also significantly influenced by dauricine treatment (15 µM), which led to a 17.38% increase and 12.30% and 5.09% decreases in G1, S, and G2 phases, respectively.

### 3.3. Dauricine Downregulates Nrf2 and Increases ROS Production

**Figure 4:**
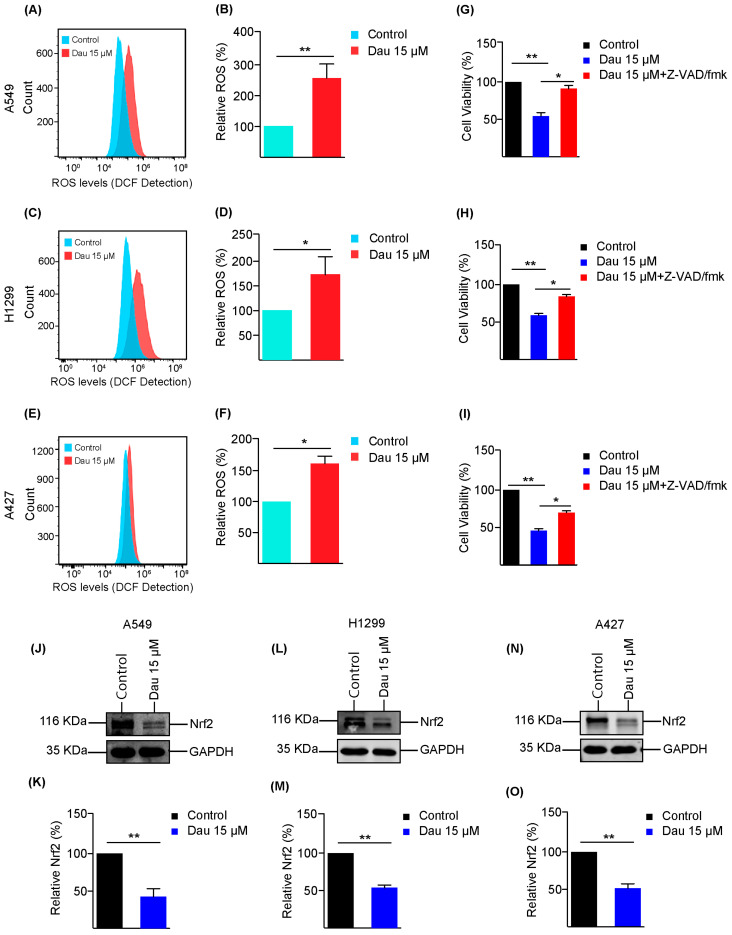
Dauricine increases the levels of ROS and reduces Nrf2 expression. (**A**) A549, (**C**) H1299, and (**E**) A427 cells were treated with dauricine at a dose of 15 µM for 24 h. Before harvesting the cells, incubation with 10 µM of DCFH-DA for 30 min was conducted to examine the ROS contents in each cell line with a flow cytometer and column charts representing the quantification of ROS in (**B**) A549, (**D**) H1299, and (**F**) A427. Cultured LUAD cells subjected to the treatment of dauricine (15 µM) with or without 10 µM of Z-VAD/fmk for 24 h were incubated with MTT solution to measure growth progression in three lung cancer cell line: (**G**) A549, (**H**) H1299, and (**I**) A427. LUAD cells were exposed to dauricine at a concentration of 15 µM for 24 h and lysates of (**J**) A549, (**L**) H1299, (**N**) A427 cells were collected to observe protein expression by Western blotting with indicated antibodies. The quantification was performed with Image Studio software and was shown in column charts for (**K**) A549, (**M**) H1299, and (**O**) A427. All error bars represent the standard error of the mean (n = 3), and * *p* < 0.05, ** *p* < 0.01.

Considering the evidence that reactive oxygen species (ROS) production was facilitated by external stimuli or inhibitors frequently causing cellular damage to suppress cell growth, we next investigated the effects of dauricine treatment on intracellular ROS levels in LUAD cells [53]. We found significantly enhanced production of ROS in A549 cells along with the quantification shown in Figure 4A,B. A similar outcome of ROS production was measured in elevated quantities after treatment with 15 µM of dauricine as compared to control cells of H1299 (Figure 4C,D). A427 cells depicted a moderate elevation of ROS levels that were also statistically significant (Figure 4E,F). Moreover, we further confirmed ROS production by flow cytometry by including *N*-Acetyl-L-cysteine (NAC) and H_2_O_2_ as controls [54]. As illustrated in Supplementary Figure S1A–F, NAC effectively blocked ROS production, while H_2_O_2_ treatment led to increased ROS, as expected. Additionally, we also confirmed the effects of ROS production on cell proliferation by MTT assays on different cell lines with NAC and H_2_O_2_ treatment (Supplementary Figure S1G–I).

Furthermore, there is a defense system accessible within the cells that protects the cells from ROS stresses, and the major regulator of this protection is the transcription factor Nrf2. The Nrf2 expression is upregulated to produce an antioxidant response element to maintain the equilibrium within the cell [55]. We observed significant downregulation of Nrf2 expression with dauricine treatment at 15 µM of dosage in A549 (Figure 4J,K), H1299 (Figure 4L,M), and A427 (Figure 4N,O), as shown in Figure 4. Oxidative stress amplification is one of the main reasons for the execution of programmed cell death [56]. It has also been reported that Nrf2 is the key regulator in iron and lipid peroxidation-dependent cell death, known as ferroptosis [56]; we tested the combination of dauricine (15 µM) with RSL3 (ferroptosis inducer) on A549 and H1975 cells related to lung adenocarcinoma (Supplementary Figures S2 and S3). This combination has been proven to primarily enhance apoptosis rather than ferroptosis, by observing the effects of the apoptosis inhibitor (Z-VAD/fmk) and ferroptosis inhibitor (Fer-1). The growth of A549, H1299, and A427 cells was restored significantly with Z-VAD/fmk, as shown by histograms in Figure 4G–I, respectively.

### 3.4. Dauricine Triggers Apoptosis in LUAD

**Figure 5:**
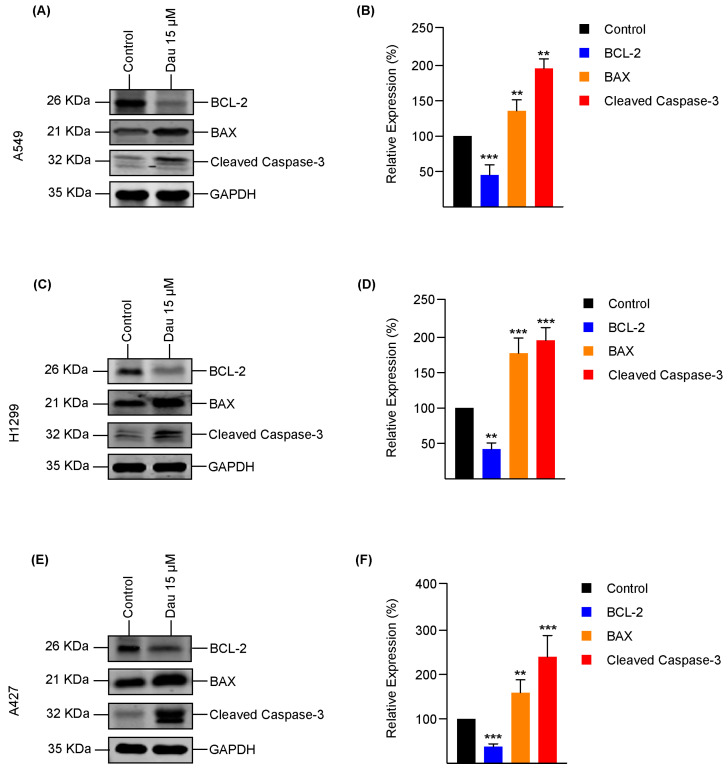
Dauricine alters the expression of apoptosis-related proteins. (**A**) A549, (**C**) H1299, and (**E**) A427 cells were treated with 15 µM of dauricine prior to cell lysis and immunoblotting was performed with designated antibodies, and GAPDH blots display loading control. Column charts for (**B**) A549, (**D**) H1299, and (**F**) A427 represent the quantification of protein levels. All error bars represent the standard error of the mean (n = 3), and ** and *** indicating *p* < 0.01 and *p* < 0.001 respectively.

We conducted further investigation into the effects of dauricine at a concentration of 15 µM on various lung cancer cell lines (A549, H1299, and A427). This study specifically examined the expression of apoptotic markers based on our observations of growth rescue by the apoptosis inhibitor Z-VAD/fmk. ROS-dependent cell apoptosis is known to be regulated by the B-cell Lymphoma 2 (Bcl-2) protein family [57]. These Bcl-2 family members are characterized on the basis of Bcl-2 homology (BH) domains [58,59]. Upon migration to the mitochondrial membrane, they trigger the activation of pro-apoptotic proteins containing BH1-BH3 domains (e.g., BAX, Bak) while inhibiting the pro-survival proteins that possess BH1-BH4 domains (e.g., Bcl-XL, Bcl-2, Mcl-1) [60,61]. Therefore, we performed immunoblotting to examine the expression of Bcl-2 and BAX following dauricine treatment, along with the apoptosis marker cleaved caspase-3. Our results revealed a significant downregulation of the Bcl-2 protein, which is known to inhibit apoptosis and promote cell survival, after treatment with dauricine. This downregulation is illustrated in the immunoblots presented in Figure 5A,C,E, showcasing its effects across different lung adenocarcinoma cell types. In contrast, we also noted a marked upregulation of BAX (Bcl-2-associated X protein) along with cleaved caspase-3, which is a vital executioner protein in the apoptotic cascade. The combined effects of increased BAX levels and activated caspase-3 suggest a robust shift toward promoting cell death in the treated lung cancer cells. These findings are represented with quantification and statistical analysis in Figure 5A–F.

### 3.5. Dauricine Diminishes the Progression of LUAD In Vivo

**Figure 6:**
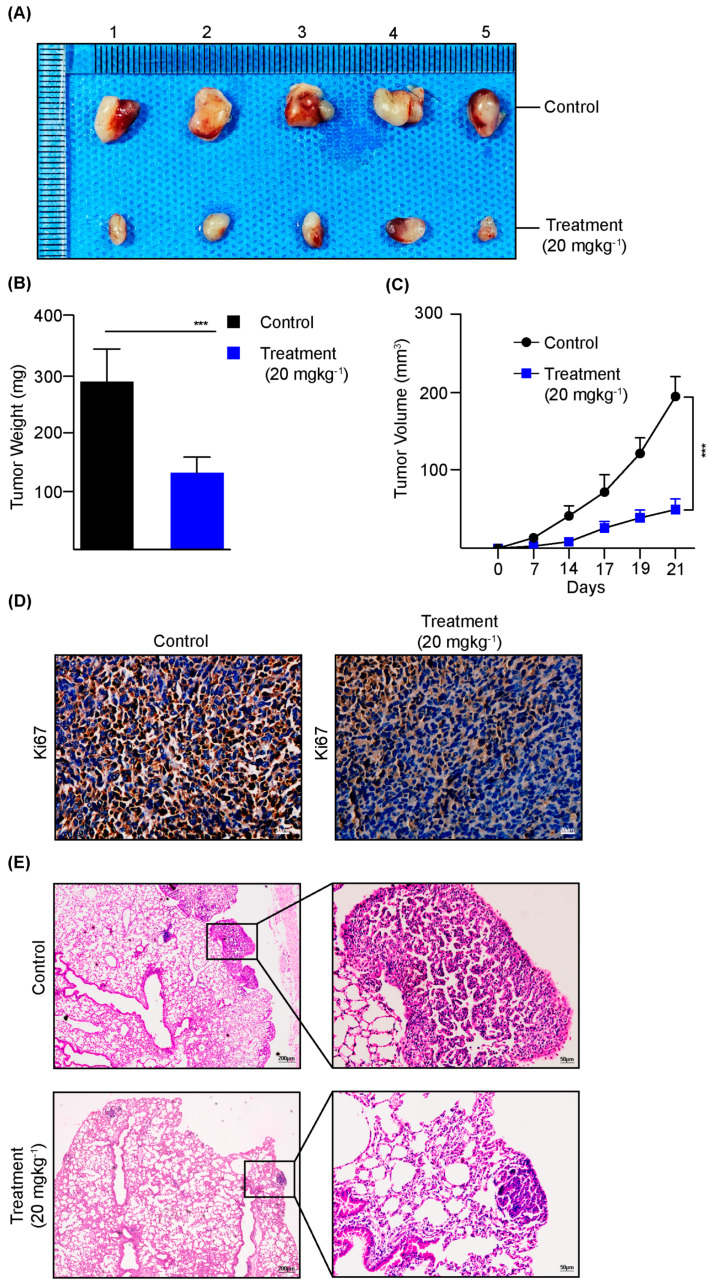
Dauricine inhibits the progression of LUAD in mice models. (**A**) LLC cells were inoculated subcutaneously into wild-type C57BL/6 mice for a syngeneic LUAD model. The image presents resected syngeneic tumors from two comparative groups as indicated. (**B**,**C**) The weights and volumes of tumors from two groups (control and treatment of dauricine at 20 mg kg^−1^) were analyzed throughout the experiment and plotted. All error bars represent the standard error of the mean (n = 10), and *** indicating *p* < 0.001. (**D**) Representative images show the immunohistochemical staining of Ki67 in syngeneic tissue sections from control and treatment groups. Scale bar = 20 µm. (**E**) Representative images show H & E staining of lung tissues from control and treatment groups of C57BL/6 transgenic mice. Scale bar = 50 µm.

To determine the efficacy of dauricine on lung cancer growth in vivo, we utilized Lewis lung carcinoma cells and developed syngeneic tumor models in C57BL/6 mice. The mice were distributed randomly into two groups and treated with dauricine (20 mgkg^−1^) or PBS for control. We found significant differences between the growth of syngeneic tumor tissues in controls as compared to the group, which is shown in Figure 6A,C. Next, we performed immunohistochemistry to evaluate the proliferation inhibition by staining with Ki67 antibody and observed substantial growth hinderance in tissues from the treatment group, as depicted in Figure 6D. Afterwards, we used another orthotopic LUAD mouse model to investigate the effects of dauricine, in which transgenic mice (*KRAS^G12D^*) were intratracheally injected with AAV2/6-CMV/Cre-ZsGreen to induce LUAD carcinogenesis before treatment with dauricine (20 mgkg^−1^) for 45 days. LUAD tumor development was assessed by performing H & E histological staining using resected lung tissues, from which we observed that the development of LUAD was drastically inhibited by dauricine, as shown in Figure 6E.

## 4. Discussion

Dauricine, a bioactive compound extracted from the rhizome of *Menispermum dauricum*, has demonstrated tremendous promising pharmacological effects in clinical studies. It has been used as a traditional Chinese medicine remedy for treating inflammatory disorders. Numerous studies have investigated the inhibitory effect of dauricine on tumor cell growth. In a study by Zhang et al., dauricine inhibited the viability of renal cell carcinoma cell lines through the induction of cell cycle arrest at the G_0_/G_1_ phase and apoptosis [45,62]. Dauricine suppressed the angiogenesis in human breast cancer by suppressing the expression of vascular endothelial growth factor and the accumulation of hypoxia-inducible factor 1α (HIF-1α) protein [63]. All these findings suggest that dauricine could be a promising therapeutic agent for the treatment of cancer. Therefore, this study focused on the antitumor effects of dauricine in the context of LUAD.

In the current study, the effects of dauricine on four NSCLC cancer cell lines (A549, H1299, LLC, and A427 cells) alongside an LUAD syngeneic tumor model and transgenic *KRAS^G12D^*-driven orthotopic LUAD model were investigated. Additionally, the influence on cellular ROS and Nrf2 expression as an underlying molecular mechanism was also investigated. Our findings underscore the significant anti-cancer effects of dauricine on LUAD cells, offering insights into its mechanisms of action, which involve the modulation of oxidative stress, downregulation of Nrf2, and activation of the apoptotic pathway. Therefore, these results reveal the roles of ROS and Nrf2 as critical regulators in dauricine-induced suppression of LUAD cell growth and survival, while highlighting dauricine as a promising therapeutic candidate for LUAD treatment.

Our results showed that in vitro treatment with two doses of dauricine, i.e., 5 and 15 µM, significantly reduced cell viability and proliferation, leading to decreases in the colony sizes in four different cell lines of LUAD. Cell proliferation is dependent on the progression of the cell cycle, which includes G1, S, and G2/M stages that confine the growth and propagation of cancer cells [64]. This has previously been reported regarding the regulatory effects of dauricine on cell cycle and cell death in renal cell carcinoma [45]. This study consistently shows that dauricine caused cell cycle arrest at the G_0_/G_1_ phase in LUAD cells. Furthermore, our findings indicate that dauricine significantly increased the production of ROS in a dose-dependent manner, with high induction occurring with a 15 µM concentration, which is in line with a previous study [65]. ROS are critical signaling molecules in cancer biology, where their regulated production is vital for cellular proliferation and survival. However, excessive ROS can cause oxidative damage to lipids, proteins, and DNA, leading to mitochondrial dysfunction and apoptosis [46,66]. The ROS-mediated cytotoxicity induced by dauricine is likely the reason for observed inhibition of cell proliferation, migration, and colony formation. Moreover, the differing sensitivity to dauricine seen across various lung adenocarcinoma cell lines may be due to the varying baseline levels of ROS in these cells [45,63,65,66]. These results support the hypothesis that cancer cells, which generally have elevated basal levels of ROS, are susceptible to additional oxidative stress caused by agents like dauricine [66].

Notably, Nrf2 plays a vital role in regulating oxidative stress and ferroptosis, a form of regulated cell death triggered by lipid peroxidation and excessive ROS. This suggests that the interplay between Nrf2 signaling and ROS production might contribute to both ferroptosis and the apoptotic effects of dauricine [64]. The apoptotic pathway is closely associated with mitochondrial dysfunction and oxidative stress. The production of ROS induced by dauricine could destabilize mitochondrial membranes, leading to the release of cytochrome c and the subsequent activation of caspase cascade. The involvement of apoptosis in the effects of dauricine was confirmed by the partial reversal of its growth-inhibitory actions by a pan-caspase inhibitor, Z-VAD/fmk [67]. Additionally, this study found that dauricine induced cell cycle arrest at the G1 phase in lung adenocarcinoma cell lines, suggesting that it can impede cell cycle progression, which may also link to apoptosis. The varying patterns of cell cycle arrest observed among the cell lines further emphasize the heterogeneity in cellular responses to dauricine, potentially linked to differences in ROS levels and the baseline expression of the transcription factor Nrf2 [45,63,67].

Importantly, the in vivo treatment with a 20 mgkg^−1^ dosage of dauricine was investigated with syngeneic C57BL/6 and transgenic *KRAS^G12D^* mice models. The in vivo efficacy of dauricine in reducing tumor burden in both syngeneic and transgenic mouse models enhances its translational potential. The significant reduction in tumor sizes, Ki67 staining, and lung adenocarcinoma development in mouse models demonstrates dauricine’s capability to inhibit tumor proliferation and progression with in vivo systems [63,67].

## 5. Conclusions

The coordination between dauricine-induced ROS elevation, downregulation of Nrf2, and activation of apoptotic pathway is fundamental to its anti-cancer effects in LUAD. By disrupting the redox balance and the antioxidant defenses, dauricine selectively targets the weaknesses of cancer cells, resulting in cell cycle arrest and apoptosis. These findings strongly support the need for further preclinical and clinical investigation of dauricine as a potential therapeutic agent for LUAD, particularly regarding its significant effects in suppressing LUAD tumorigenesis in different animal models.

## Data Availability

All data generated during this study are included in this published article.

## Supplementary Materials

The following supporting information can be downloaded at: https://www.mdpi.com/article/10.3390/cells14100698/s1, Figure S1: Dauricine induced ROS increases and reduced cell viability are reversed by NAC; Figure S2: Characterization of the effects of dauricine with ferroptosis inducer RSL3 in A549 cells; Figure S3: Characterization of the effects of dauricine with ferroptosis inducer RSL3 in H1975 cells.

## References

[1] Kodaz C.E. (2022). Lung Cancer Risk factors—A review article. Eurasian J. Med. Adv..

[2] Adler R.H. (1975). Lung cancer: A review. West Indian Med. J..

[3] Zhao J., Lin X., Meng D., Zeng L., Zhuang R., Huang S., Lv W., Hu J. (2020). Nrf2 Mediates Metabolic Reprogramming in Non-Small Cell Lung Cancer. Front. Oncol..

[4] Mustafa M., Azizi A.R.J., IIIzam E.L., Nazirah A., Sharifa S., Abbas S.A. (2016). Lung Cancer: Risk Factors, Management, And Prognosis. IOSR J. Dent. Med. Sci..

[5] Sung H., Ferlay J., Siegel R.L., Laversanne M., Soerjomataram I., Jemal A., Bray F. (2021). Global Cancer Statistics 2020: GLOBOCAN Estimates of Incidence and Mortality Worldwide for 36 Cancers in 185 Countries. CA A Cancer J. Clin..

[6] Deo S.V.S., Sharma J., Kumar S. (2022). GLOBOCAN 2020 Report on Global Cancer Burden: Challenges and Opportunities for Surgical Oncologists. Ann. Surg. Oncol..

[7] de Groot P.M., Wu C.C., Carter B.W., Munden R.F. (2018). The epidemiology of lung cancer. Transl. Lung Cancer Res..

[8] Gridelli C., Rossi A., Carbone D.P., Guarize J., Karachaliou N., Mok T., Petrella F., Spaggiari L., Rosell R. (2015). Non-small-cell lung cancer. Nat. Rev. Dis. Primers.

[9] Singh A., Daemen A., Nickles D., Jeon S.-M., Foreman O., Sudini K., Gnad F., Lajoie S., Gour N., Mitzner W. (2021). NRF2 Activation promotes aggressive lung cancer and associates with poor clinical outcomes. Clin. Cancer Res..

[10] Torre L.A., Bray F., Siegel R.L., Ferlay J., Lortet-Tieulent J., Jemal A. (2015). Global cancer statistics, 2012. CA Cancer J. Clin..

[11] Siegel R.L., Miller K.D., Jemal A. (2018). Cancer statistics, 2018. CA Cancer J. Clin..

[12] Molina J.R., Yang P., Cassivi S.D., Schild S.E., Adjei A.A. (2008). Non-small cell lung cancer: Epidemiology, risk factors, treatment, and survivorship. Mayo Clin. Proc..

[13] Zhang J., Fujimoto J., Zhang J., Wedge D.C., Song X., Zhang J., Seth S., Chow C.W., Cao Y., Gumbs C. (2014). Intratumor heterogeneity in localized lung adenocarcinomas delineated by multiregion sequencing. Science.

[14] Lachgar A., Tazi M.A., Afif M., Er-Raki A., Kebdani T., Benjaafar N. (2016). Le cancer du poumon: Incidence et survie à Rabat, Maroc. Rev. D’epidemiologie Et De Sante Publique.

[15] Wang M., Herbst R.S., Boshoff C. (2021). Toward personalized treatment approaches for non-small-cell lung cancer. Nat. Med..

[16] Herbst R.S., Morgensztern D., Boshoff C. (2018). The biology and management of non-small cell lung cancer. Nature.

[17] Tan C.-S., Gilligan D., Pacey S. (2015). Treatment approaches for EGFR-inhibitor-resistant patients with non-small-cell lung cancer. Lancet Oncol..

[18] Rachiglio A.M., Abate R.E., Sacco A., Pasquale R., Fenizia F., Lambiase M., Morabito A., Montanino A., Rocco G., Romano C. (2016). Limits and potential of targeted sequencing analysis of liquid biopsy in patients with lung and colon carcinoma. Oncotarget.

[19] Pelos G., Riester M., Pal J., Myacheva K., Moneke I., Rotondo J.C., Lübbert M., Diederichs S. (2024). Fast proliferating and slowly migrating non-small cell lung cancer cells are vulnerable to decitabine and retinoic acid combinatorial treatment. Int. J. Cancer.

[20] Cukier P., Santini F.C., Scaranti M., Hoff A.O. (2017). Endocrine side effects of cancer immunotherapy. Endocr.-Relat. Cancer.

[21] Kroschinsky F., Stölzel F., von Bonin S., Beutel G., Kochanek M., Kiehl M., Schellongowski P. (2017). New drugs, new toxicities: Severe side effects of modern targeted and immunotherapy of cancer and their management. Crit. Care.

[22] Skoulidis F., Heymach J.V. (2019). Co-occurring genomic alterations in non-small-cell lung cancer biology and therapy. Nat. Rev. Cancer.

[23] Luo J., Ostrem J., Pellini B., Imbody D., Stern Y., Solanki H.S., Haura E.B., Villaruz L.C. (2022). Overcoming KRAS-Mutant Lung Cancer, American Society of Clinical Oncology Educational Book. American Society of Clinical Oncology. Annu. Meet..

[24] Campbell J.D., Alexandrov A., Kim J., Wala J., Berger A.H., Pedamallu C.S., Shukla S.A., Guo G., Brooks A.N., Murray B.A. (2016). Distinct patterns of somatic genome alterations in lung adenocarcinomas and squamous cell carcinomas. Nat. Genet..

[25] Canon J., Rex K., Saiki A.Y., Mohr C., Cooke K., Bagal D., Gaida K., Holt T., Knutson C.G., Koppada N. (2019). The clinical KRAS(G12C) inhibitor AMG 510 drives anti-tumour immunity. Nature.

[26] Newman D.J., Cragg G.M. (2020). Natural Products as Sources of New Drugs over the Nearly Four Decades from 01/1981 to 09/2019. J. Nat. Prod..

[27] Dilly J., Hoffman M.T., Abbassi L., Li Z., Paradiso F., Parent B.D., Hennessey C.J., Jordan A.C., Morgado M., Dasgupta S. (2024). Mechanisms of resistance to oncogenic KRAS inhibition in pancreatic cancer. Cancer Discov..

[28] Drosten M., Barbacid M. (2022). Targeting KRAS mutant lung cancer: Light at the end of the tunnel. Mol. Oncol..

[29] Martínez-Reyes I., Chandel N.S. (2021). Cancer metabolism: Looking forward, Nature Reviews. Cancer.

[30] Vander Heiden M.G., DeBerardinis R.J. (2017). Understanding the Intersections between Metabolism and Cancer Biology. Cell.

[31] Chio I.I.C., Tuveson D.A. (2017). ROS in Cancer: The Burning Question. Trends Mol. Med..

[32] Lee A.C., Fenster B.E., Ito H., Takeda K., Bae N.S., Hirai T., Yu Z.X., Ferrans V.J., Howard B.H., Finkel T. (1999). Ras proteins induce senescence by altering the intracellular levels of reactive oxygen species. J. Biol. Chem..

[33] Li Y., Zhang X., Wang Z., Li B., Zhu H. (2023). Modulation of redox homeostasis: A strategy to overcome cancer drug resistance. Front. Pharmacol..

[34] Harris I.S., DeNicola G.M. (2020). The Complex Interplay between Antioxidants and ROS in Cancer. Trends Cell Biol..

[35] Tan C., Smolenski R., Harhun M., Patel H., Ahmed S., Wanisch K., Yáñez-Muñoz R., Baines D. (2012). AMP-activated protein kinase (AMPK)-dependent and -independent pathways regulate hypoxic inhibition of transepithelial Na+ transport across human airway epithelial cells. Br. J. Pharmacol..

[36] Tamari S., Menju T., Toyazaki T., Miyamoto H., Chiba N., Noguchi M., Ishikawa H., Miyata R., Kayawake H., Tanaka S. (2022). Nrf2/p-Fyn/ABCB1 axis accompanied by p-Fyn nuclear accumulation plays pivotal roles in vinorelbine resistance in non-small cell lung cancer. Oncol. Rep..

[37] Deen A.J., Adinolfi S., Härkönen J., Patinen T., Liu X., Laitinen T., Takabe P., Kainulainen K., Pasonen-Seppänen S., Gawriyski L.M. (2024). Oncogenic KEAP1 mutations activate TRAF2-NFκB signaling to prevent apoptosis in lung cancer cells. Redox Biol..

[38] Singh A., Misra V., Thimmulappa R.K., Lee H., Ames S., O Hoque M., Herman J.G., Baylin S.B., Sidransky D., Gabrielson E. (2006). Dysfunctional KEAP1-NRF2 interaction in non-small-cell lung cancer. PLoS Med..

[39] Singh A., Venkannagari S., Oh K.H., Zhang Y.-Q., Rohde J.M., Liu L., Nimmagadda S., Sudini K., Brimacombe K.R., Gajghate S. (2016). Small Molecule Inhibitor of NRF2 Selectively Intervenes Therapeutic Resistance in KEAP1-Deficient NSCLC Tumors. ACS Chem. Biol..

[40] Zhang J., Wang X., Vikash V., Ye Q., Wu D., Liu Y., Dong W. (2016). ROS and ROS-Mediated Cellular Signaling. Oxid. Med. Cell. Longev..

[41] Wang L., Pu Z., Li M., Wang K., Deng L., Chen W. (2020). Antioxidative and antiapoptosis: Neuroprotective effects of dauricine in Alzheimer’s disease models. Life Sci..

[42] Liang J., Lei P., Su X., Gao J., Ren B., Zhang Y., Ma X., Ma W. (2024). Dauricine Inhibits Non-small Cell Lung Cancer Development by Regulating PTEN/AKT/mTOR and Ras/MEK1/2/ERK1/2 Pathways in a FLT4-dependent Manner. Curr. Cancer Drug Targets.

[43] Wang J., Li Y., Zu X.B., Chen M.F., Qi L. (2012). Dauricine can inhibit the activity of proliferation of urinary tract tumor cells. Asian Pac. J. Trop. Med..

[44] Zhang Y., Fei H., Guo J., Zhang X., Wu S., Zhong L. (2019). Dauricine suppresses the growth of pancreatic cancer in vivo by modulating the Hedgehog signaling pathway. Oncol. Lett..

[45] Zhang S., Ren Y., Qiu J. (2018). Dauricine inhibits viability and induces cell cycle arrest and apoptosis via inhibiting the PI3K/Akt signaling pathway in renal cell carcinoma cells. Mol. Med. Rep..

[46] Yang Z., Li C., Wang X., Zhai C., Yi Z., Wang L., Liu B., Du B., Wu H., Guo X. (2010). Dauricine induces apoptosis, inhibits proliferation and invasion through inhibiting NF-kappaB signaling pathway in colon cancer cells. J. Cell. Physiol..

[47] Wang T., Wang D., Zhang Y., Zhang J., Sun X., Wu Y., Wang S., Zhang Y., Xu L., Kong Q. (2018). Dynasore-induced potent ubiquitylation of the exon 19 deletion mutant of epidermal growth factor receptor suppresses cell growth and migration in non-small cell lung cancer. Int. J. Biochem. Cell Biol..

[48] Zhang Y., Zhang J., Li Q., Wu Y., Wang D., Xu L., Zhang Y., Wang S., Wang T., Liu F. (2019). Cholesterol content in cell membrane maintains surface levels of ErbB2 and confers a therapeutic vulnerability in ErbB2-positive breast cancer. Cell Commun. Signal..

[49] Cheng S., Qiu Z., Zhang Z., Li Y., Zhu Y., Zhou Y., Yang Y., Zhang Y., Yang D., Zhang Y. (2025). USP39 phase separates into the nucleolus and drives lung adenocarcinoma progression by promoting GLI1 expression. Cell Commun. Signal. CCS.

[50] Zaky M.Y., Liu X., Wang T., Wang S., Liu F., Wang D., Wu Y., Zhang Y., Guo D., Sun Q. (2020). Dynasore potentiates c-Met inhibitors against hepatocellular carcinoma through destabilizing c-Met. Arch. Biochem. Biophys..

[51] Siddiqui N.Z., Rehman A.U., Yousuf W., Khan A.I., Farooqui N.A., Zang S., Xin Y., Wang L. (2022). Effect of crude polysaccharide from seaweed, Dictyopteris divaricata (CDDP) on gut microbiota restoration and anti-diabetic activity in streptozotocin (STZ)-induced T1DM mice. Gut Pathog..

[52] Zhang J., Liu S., Li Q., Shi Y., Wu Y., Liu F., Wang S., Zaky M.Y., Yousuf W., Sun Q. (2020). The deubiquitylase USP2 maintains ErbB2 abundance via counteracting endocytic degradation and represents a therapeutic target in ErbB2-positive breast cancer. Cell Death Differ..

[53] Redza-Dutordoir M., Averill-Bates D.A. (2016). Activation of apoptosis signalling pathways by reactive oxygen species. Biochim. Et Biophys. Acta.

[54] Chen F., Xiao M., Hu S., Wang M. (2024). Keap1-Nrf2 pathway: A key mechanism in the occurrence and development of cancer. Front. Oncol..

[55] Yan R., Lin B., Jin W., Tang L., Hu S., Cai R. (2023). NRF2, a Superstar of Ferroptosis. Antioxidants.

[56] Lavrik I.N., Golks A., Krammer P.H. (2005). Caspases: Pharmacological manipulation of cell death. J. Clin. Investig..

[57] Brunelle J.K., Letai A. (2009). Control of mitochondrial apoptosis by the Bcl-2 family. J. Cell Sci..

[58] Kaloni D., Diepstraten S.T., Strasser A., Kelly G.L. (2023). BCL-2 protein family: Attractive targets for cancer therapy. Apoptosis Int. J. Program. Cell Death.

[59] Shamas-Din A., Kale J., Leber B., Andrews D.W. (2013). Mechanisms of action of Bcl-2 family proteins. Cold Spring Harb. Perspect. Biol..

[60] Czabotar P.E., Lessene G., Strasser A., Adams J.M. (2014). Control of apoptosis by the BCL-2 protein family: Implications for physiology and therapy, Nature Reviews. Mol. Cell Biol..

[61] Zhong L.-L., Zhou Z.-G., Zhang C.-Y., Fei H.-X., Bai Y. (2015). Phenolic alkaloids from Menispermum dauricum inhibits BxPC-3 pancreatic cancer cells by blocking of Hedgehog signaling pathway. Pharmacogn. Mag..

[62] Tang X.-D., Zhou X., Zhou K.-Y. (2009). Dauricine inhibits insulin-like growth factor-I-induced hypoxia inducible factor 1α protein accumulation and vascular endothelial growth factor expression in human breast cancer cells. Acta Pharmacol. Sin..

[63] Chan C.K., Chan G., Awang K., Kadir H.A. (2016). Deoxyelephantopin from Elephantopus scaber Inhibits HCT116 Human Colorectal Carcinoma Cell Growth through Apoptosis and Cell Cycle Arrest. Molecules.

[64] Huang X.-H., Yan X., Zhang Q.-H., Hong P., Zhang W.-X., Liu Y.-P., Xu W.W., Li B., He Q.-Y. (2020). Direct targeting of HSP90 with daurisoline destabilizes β-catenin to suppress lung cancer tumorigenesis. Cancer Lett..

[65] Chen K.-Q., Wang S.-Z., Lei H.-B., Liu X. (2024). Dauricine: Review of Pharmacological Activity. Drug Des. Dev. Ther..

[66] Chen Y., Jiang Z., Li X. (2024). New insights into crosstalk between Nrf2 pathway and ferroptosis in lung disease. Cell Death Dis..

[67] Rezaei P.F., Fouladdel S., Cristofanon S., Ghaffari S.M., Amin G.R., Azizi E. (2011). Comparative cellular and molecular analysis of cytotoxicity and apoptosis induction by doxorubicin and Baneh in human breast cancer T47D cells. Cytotechnology.

