# Supplementary material for: Dauricine Impedes the Tumorigenesis of Lung Adenocarcinoma by Regulating Nrf2 and Reactive Oxygen Species
Source: Cells. 2025 May 12;14(10):698. doi: 10.3390/cells14100698 (PMC12109956; doi:10.3390/cells14100698)
Supplement: Supplementary file 1 [file cells-14-00698-s001.zip › cells-3554803-supplementary.pdf]

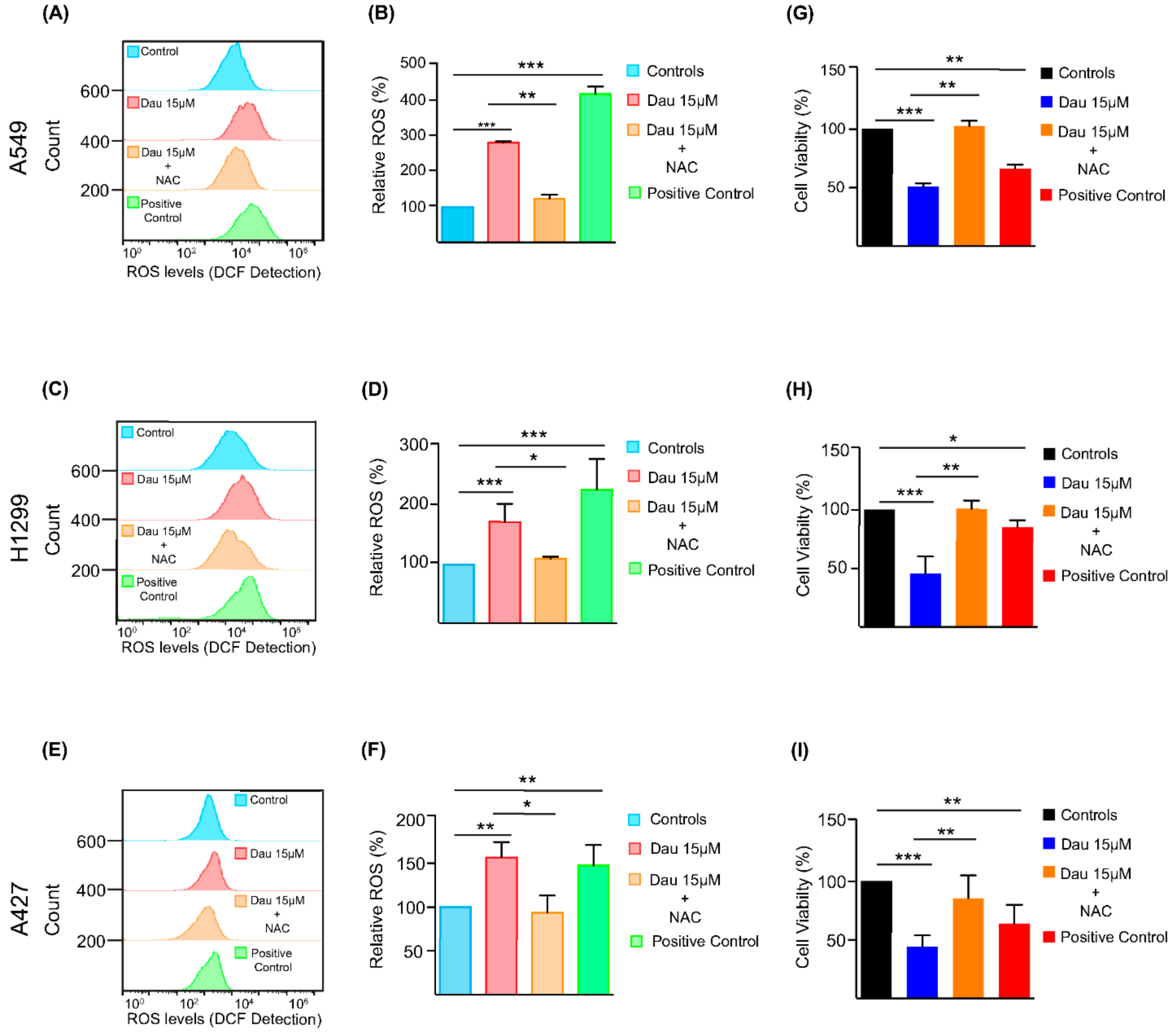

Supplementary Figure S1. Dauricine induced ROS increases and reduced cell viability are reversed by NAC. **(A)** A549 **(C)** H1299 **(E)** A427 cells were treated with DMSO as control, dauricine with or without N-Acetyl-L-cysteine (NAC) at 2 mM, compared with positive control H_2O_2 at 1 mM. Cellular ROS levels were measured by flow cytometry in indicated lung adenocarcinoma cell lines. **(B)** A549 **(D)** H1299 and **(F)** A427 charts depict the quantification of ROS production. **(G)** A549, **(H)** H1299, **(I)** A427 cells were processed for MTT assays following indicated treatments to examine cell viability. All error bars represent the standard error of the mean (n = 3), and * indicates $p<0.05$, ** $p < 0.01$, *** $p < 0.001$.

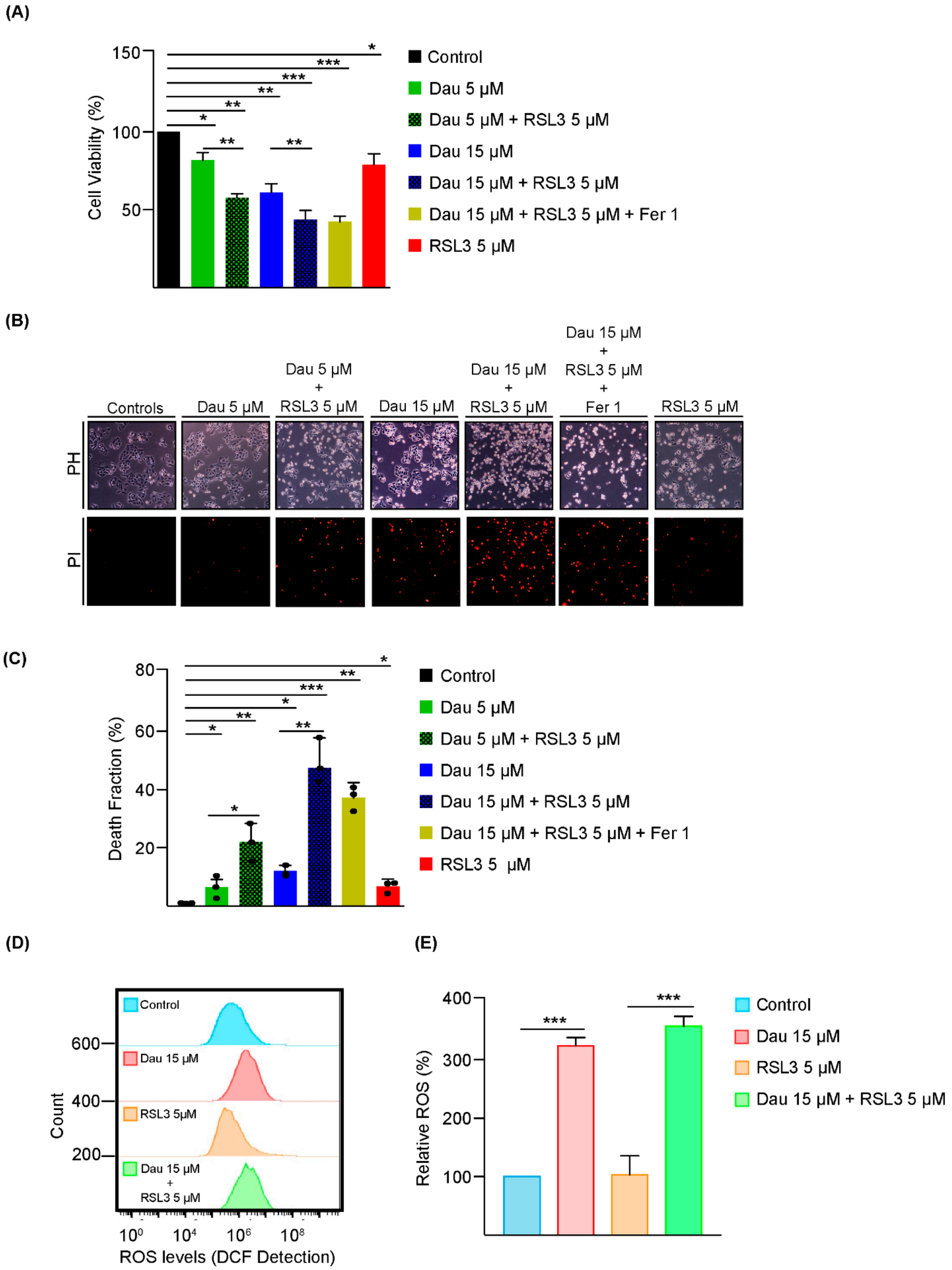

(A)
150
100
50
Cell Viability (%)
*
***
***
**
**
*
**
**
Control
Dau 5 μM
Dau 5 μM + RSL3 5 μM
Dau 15 μM
Dau 15 μM + RSL3 5 μM
Dau 15 μM + RSL3 5 μM + Fer 1
RSL3 5 μM
(B)
Controls
Dau 5 μM
Dau 5 μM + RSL3 5 μM
Dau 15 μM
Dau 15 μM + RSL3 5 μM
Dau 15 μM + RSL3 5 μM + Fer 1
RSL3 5 μM
PH
PI
(C)
80
60
40
20
Death Fraction (%)
*
**
***
*
**
*
**
*
Control
Dau 5 μM
Dau 5 μM + RSL3 5 μM
Dau 15 μM
Dau 15 μM + RSL3 5 μM
Dau 15 μM + RSL3 5 μM + Fer 1
RSL3 5 μM
(D)
Control
Dau 15 μM
RSL3 5μM
Dau 15 μM + RSL3 5 μM
600
400
200
Count
10⁰ 10⁴ 10⁶ 10⁸
ROS levels (DCF Detection)
(E)
400
300
200
100
Relative ROS (%)
***
***
Control
Dau 15 μM
RSL3 5 μM
Dau 15 μM + RSL3 5 μM

Supplementary Figure S2. Characterization of the effects of dauricine with ferroptosis inducer RSL3 in A549 cells. **(A)** A549 cell proliferation was examined with indicated treatments for 24 h via MTT assays. **(B)** Representative images show the propidium iodide staining performed to investigate cell death following indicated treatments for 24 h. **(C)** Quantification of PI-stained cells in B. **(D)** A549 cells were exposed to indicated treatments of dauricine with or without RSL3 (5 μM) to observe ROS production.

(E) ROS quantification from D. All error bars represent the standard error of the mean (n = 3), and * indicates $p<0.05$, ** $p < 0.01$, *** $p < 0.001$.

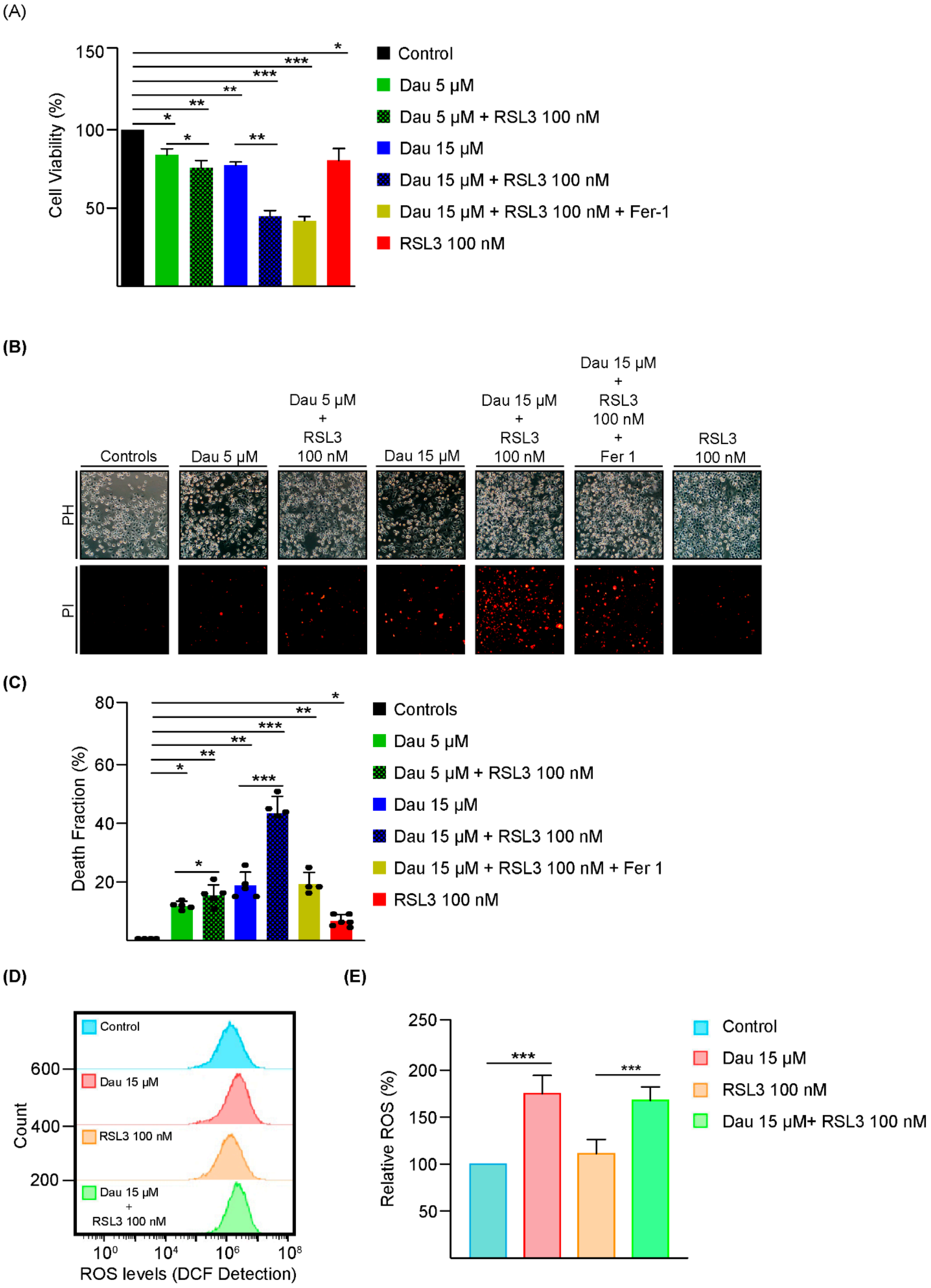

(A)
150
100
50
Cell Viability (%)
*
***
***
**
**
*
*
**
Control
Dau 5 μM
Dau 5 μM + RSL3 100 nM
Dau 15 μM
Dau 15 μM + RSL3 100 nM
Dau 15 μM + RSL3 100 nM + Fer-1
RSL3 100 nM
(B)
Controls
Dau 5 μM
Dau 5 μM + RSL3 100 nM
Dau 15 μM
Dau 15 μM + RSL3 100 nM
Dau 15 μM + RSL3 100 nM + Fer 1
RSL3 100 nM
PH
PI
(C)
80
60
40
20
Death Fraction (%)
*
**
***
**
**
*
***
*
Controls
Dau 5 μM
Dau 5 μM + RSL3 100 nM
Dau 15 μM
Dau 15 μM + RSL3 100 nM
Dau 15 μM + RSL3 100 nM + Fer 1
RSL3 100 nM
(D)
Control
Dau 15 μM
RSL3 100 nM
Dau 15 μM + RSL3 100 nM
600
400
200
Count
10⁰ 10⁴ 10⁶ 10⁸
ROS levels (DCF Detection)
(E)
250
200
150
100
50
Relative ROS (%)
***
***
Control
Dau 15 μM
RSL3 100 nM
Dau 15 μM+ RSL3 100 nM

Supplementary Figure S3. Characterization of the effects of dauricine with ferroptosis inducer RSL3 in H1975 cells. **(A)** H1975 cell proliferation was examined with indicated treatments for 24 h via MTT assays. **(B)** Representative images show the propidium iodide staining performed to investigate cell death following indicated treatments for 24 h. **(C)** Quantification of PI-stained cells in B. **(D)** H1975 cells were exposed to indicated treatments of dauricine with or without RSL3 (100 nM) to observe ROS production. **(E)** ROS quantification from D. All error bars represent the standard error of the mean (n = 3), and * indicates $p<0.05$, ** $p < 0.01$, *** $p < 0.001$.
